# Uptake, Elimination and Metabolism of Brominated Dibenzofurans in Mice

**DOI:** 10.3390/toxics12090656

**Published:** 2024-09-07

**Authors:** Nguyen Minh Tue, Eiki Kimura, Fumihiko Maekawa, Akitoshi Goto, Naoto Uramaru, Tatsuya Kunisue, Go Suzuki

**Affiliations:** 1 Center for Marine Environmental Studies (CMES), Ehime University, 2-5 Bunkyo-cho, Matsuyama 790-8577, Japan; goto.akitoshi.xn@ehime-u.ac.jp (A.G.);; 2 Health and Environmental Risk Division, National Institute for Environmental Studies (NIES), 16-2 Onogawa, Tsukuba 305-8506, Japan; kimuraei@u-fukui.ac.jp (E.K.);; 3 Department of Environmental Health, School of Medical Sciences, University of Fukui, 23-3 Matsuoka Shimoaizuki, Eiheiji 910-1193, Japan; 4 Division of Pharmaceutical Health Biosciences, Nihon Pharmaceutical University, 10281 Komuro, Ina-machi, Kitaadachi, Saitama 362-0806, Japan; 5 School of Health and Social Services, Center for University-wide Education, Saitama Prefectural University, 820 San-Nomiya, Koshigaya, Saitama 343-8540, Japan; 6 Material Cycles Division, NIES, 16-2 Onogawa, Tsukuba 305-8506, Japan; g-suzuki@nies.go.jp

**Keywords:** brominated dioxins, metabolites, mouse, tissue distribution, toxicokinetics

## Abstract

Polybrominated dibenzofurans (PBDFs) are major brominated dioxins in the environment, but information on their bioaccumulation potential and toxicokinetics is limited. This study conducted oral exposure experiments with C57BL/6J mice to investigate the uptake ratios, distribution in the liver, plasma and brain, metabolism, and elimination kinetics of four bromine/chlorine-substituted dibenzofurans (TrBDF: 2,3,8-tribromo, TeBDF: 2,3,7,8-tetrabromo, PeBDF: 1,2,3,7,8-pentabromo, TrBCDF: 2,3,7-tribromo-8-chloro) in comparison with 2,3,7,8-tetrachlorodibenzo-p-dioxin (TCDD). The hepatic uptake ratios of 2,3,7,8-substituted dibenzofurans were lower than that of TCDD (up to 84% of the administered doses) and decreased with the number of Br substitutions (42%, 33%, and 29% for TrBCDF, TeBDF, and PeBDF, respectively). The brain uptake ratios of these dibenzofurans were less than 0.05%, and the plasma-to-brain transfer ratio also decreased with the Br number. All 2,3,7,8-substituted compounds were eliminated from the liver following first-order kinetics, with half-times in the order of TrBCDF (5.6 days) < TeBDF (8.8 days) ≈ TCDD (8.7 days) < PeBDF (13 days). The non-2,3,7,8-substituted TrBDF was poorly retained in the liver (<0.01% of the dose at 1 day) and rapidly eliminated following two-phase kinetics. All dibenzofurans were metabolised into monohydroxylated products in the liver, but the contribution of this metabolic pathway to hepatic elimination was only significant for TrBDF. As the toxic effects of dioxin-like compounds are influenced by their biological persistence, the slow elimination of TrBCDF, TeBDF, and PeBDF observed in this study suggests that exposure risk of brominated dibenzofurans may be underestimated using the toxic equivalency factors of the less persistent chlorinated analogues.

## 1. Introduction

Environmental pollution by chemicals from anthropogenic activities has led to growing concerns [1,2]. Polychlorinated dibenzo-*p*-dioxins and dibenzofurans (PCDDs and PCDFs) are persistent contaminants well-known for a wide range of toxic effects including hepatotoxicity, reproductive effects [3], neurotoxicity [4], and transgenerational effects [5]. Polybrominated dibenzo-*p*-dioxins and dibenzofurans (PBDDs and PBDFs), which are bromine-substituted compounds with the same molecular skeletons as PCDDs and PCDFs, can be generated during thermal processes like their chlorinated analogues, but their formation involves abundantly available precursors such as brominated flame retardants (BFRs) rather than de novo synthesis [6,7]. PBDFs, as by-products and thermal/photolytic decomposition products of the widely used flame retardants polybrominated diphenyl ethers (PBDEs) [6,8,9], are the major environmentally occurring brominated dioxins and have been detected at higher levels than chlorinated dioxins in effluents from PBDE-handling industrial facilities [10], e-waste plastics [11,12], various environmental matrices from e-waste and end-of-life vehicle dismantling sites [13,14], and indoor dust from common households [15,16]. PBDFs can also undergo consecutive bromine-to-chlorine exchange under thermal and catalytic conditions in the presence of chlorine-containing materials, producing mixed brominated/chlorinated analogues (PXDFs) [6], preferentially bromine-rich congeners (with fewer chlorine substitutions), as observed in soils around e-waste burning areas [17] and effluents from textile facilities handling PBDEs [18]. It is thus necessary to consider potential adverse effects of brominated dioxins, especially PBDFs and PXDFs.

PCDDs and PCDFs with chlorine substitutions at positions 2,3,7,8 are known to be able to persistently activate the aryl-hydrocarbon receptor (AhR) signalling pathway, causing a wide variety of dioxin-like toxic effects [3]. Many 2,3,7,8-substituted PBDDs/PBDFs and PXDDs/PXDFs were also found to be potent AhR agonists using in vitro reporter gene assays [19,20,21], but in vivo toxicity studies with these brominated dioxins, especially dibenzofurans, remain very limited. Several studies have demonstrated that 2,3,7,8-substituted PBDFs cause effects similar to those of 2,3,7,8-tetrachlorodibenzo-*p*-dioxin (TCDD)—the most toxic among chlorinated dioxins—such as hydronephrosis, cleft palate, and impaired ultrasonic vocalisation in the mouse [22,23], as well as blue-sac disease in Japanese medaka [24]. Brominated dioxins have been included in the World Health Organization (WHO) toxic equivalency factor (TEF) scheme for risk assessment of dioxin-like compounds, and the TEF values of chlorinated dioxins were recommended as interim TEFs for their respective brominated and mixed brominated/chlorinated analogues, pending availability of additional toxicity and toxicokinetic information [25].

Bioaccumulation potential and persistence are important criteria for contaminants to be included in the TEF scheme [26]. However, only a few studies on the uptake, tissue distribution, elimination, and metabolism of PBDFs in experimental animals have been conducted [27,28,29,30]. Like TCDD, PBDFs also accumulate mainly in the liver and adipose tissue but with lower uptake ratios [27,28]. The persistence of 2,3,7,8-substituted PBDFs was found to be comparable to similarly structured chlorinated dioxins in rats, based on the hepatic elimination half-times observed for tetra- and penta-brominated PBDFs [27]. Nevertheless, direct comparison of toxicokinetic parameters is not reliable, as most data for chlorinated dioxins were obtained in exposure experiments with radioisotope-labelled standards [31,32] and derived from the residual radioactivity contributed by both unmetabolised and metabolised forms of the standards.

Based on the limitations of the available data, the present study conducted exposure experiments with C57BL/6J mice to investigate the uptake ratio, tissue distribution, and elimination kinetics of several brominated and mixed brominated/chlorinated dibenzofurans in comparison with TCDD. The C57BL/6J mouse strain was used in some studies on the toxicokinetics of chlorinated dioxins [31,32] and was found to be affected with hepatotoxicity and neurotoxicity in exposure experiments with TCDD and several brominated dibenzofurans [23,33,34]. It is therefore important to examine the hepatic uptake as well as the blood-to-brain transfer of dioxins in this strain. In the present study, chemical analysis of the administered compounds was conducted for not only the livers but also for the brains and plasma of the exposed mice. The influence of metabolism on toxicokinetic parameters was also assessed by analysis of monohydroxylated metabolites in the liver, as monooxygenation was reported to be a major metabolism pathway for PCDFs [35].

## 2. Materials and Methods

### 2.1. Chemicals for Exposure Experiments

The tested dibenzofurans included two 2,3,7,8-substituted PBDFs (TeBDF: 2,3,7,8-tetrabromodibenzofuran, and PeBDF: 1,2,3,7,8-pentabromodibenzofuran), one 2,3,7,8-substituted bromine-rich PXDFs (TrBCDF: 2,3,7-tribromo-8-chlorodibenzofuran), and one non-2,3,7,8-substituted PBDF (TrBDF: 2,3,8-tribromodibenzofuran). TrBDF, TeBDF, PeBDF, and TrBCDF were synthesised according to the methods described by Sovocool et al. [36] with purities of >98%, >98%, >77%, and >77%, respectively. TCDD (purity >98%) was purchased from Cambridge Isotope Laboratory (Andover, MA, USA). All compounds were stored in the dark at 4 °C.

### 2.2. Animals and Chemical Treatment

The animal exposure experiments were conducted according to a previously described protocol [23,33]. Male C57BL/6J mice, at the age of 8 weeks, were purchased from CLEA (Tokyo, Japan) and housed in an animal facility with the following conditions: temperature of 24 ± 1 °C, humidity of 50% ± 10%, light-dark cycle of 12/12 h (light from 7:00 to 19:00), laboratory rodent chow (CE-2) and distilled water provided ad libitum. Random groups of 5 or 6 mice were housed separately. A group of mice was administered by gavage with a single dose of the vehicle (10 mL/kg body weight (bw) of corn oil containing 0.6% *n*-nonane) (control group), and the other groups with the vehicle containing TCDD (5 groups), TrBCDF (4 groups), TeBDF (5 groups), PeBDF (4 groups), or TrBDF (4 groups). The concentration of *n*-nonane in the vehicle was found to have no observable effects in C57BL/6J mice, including effects on the delivery and litter size of pregnant mice [34]. The purity-corrected doses of TCDD, TrBCDF, TeBDF, PeBDF, and TrBDF were 3.0, 31, 45, 135, and 378 µg/kg bw, respectively. The exposure doses were chosen to achieve similar TCDD-equivalent doses for all exposed groups and were derived on a molar concentration basis from the interim TEF values [25] for TrBCDF, TeBDF, and PeBDF (0.1, 0.1, and 0.03, respectively). The dose of TrBDF was set 100 times higher than that of TCDD on a molar basis. Only male adult mice were used to exclude sexual cycle effects. The animals were sacrificed at 1, 3, 7, and 28 days after administration (one exposed group for each test compound and each time point), and at 56 days (one TCDD- and one TeBDF-exposed group) to harvest their livers, brains, and blood. Blood samples were drawn from the heart of the mice under anaesthesia with a mixture of anaesthetic agents containing medetomidine hydrochloride (Domitor^®^, Nippon Zenyaku Kogyo, Tokyo, Japan), midazolam (Midazolam Sandoz^®^, Sandoz, Tokyo, Japan), and butorphanol (Vetorphale^®^, Meiji Seika Pharma, Tokyo, Japan). After blood collection, plasma components were separated by centrifugation with heparin. The livers and brains of the decapitated mice were quickly collected, and then all the tissue samples were stored at −80 °C until analysis. The numbers of individuals in different groups and their body and tissue weights are given in Table S1.

### 2.3. Chemical Analysis

The tissue samples were pooled for each exposure group and time point (5–6 samples per pool), and the pooled samples were analysed based on previous analytical methods for PCBs, PBDEs, and their hydroxylated derivatives [37,38], with slight modifications. Briefly, the liver (4.2–8.4 g per pool) and brain (2.3–2.8 g per pool) samples were freeze-dried and then extracted with a tert-butyl methyl ether (tBME)/hexane mixture (1:1 v/v) using a rapid solvent extractor (SE-100, Mitsubishi Chemical Analytech, Japan). The plasma samples (1.0–2.0 g per pool) were denatured with 2-propanol and 6 M HCl, and then liquid–liquid extracted with tBME/hexane. Before extraction, the samples were spiked with ^13^C_12_-labelled TCDD and OH-PCBs (control and TCDD-exposed groups), or ^13^C_12_-labelled TeBDF and 6-OH-BDE-47 (control and other exposed groups) as surrogates. After extraction, the extracts were solvent-exchanged into hexane and then partitioned with potassium hydroxide in 50% ethanol/water to separate hydroxylated metabolites (phenolate form in alkaline phase) from the parent compounds (organic phase). The organic phase was cleaned up using gel-permeation chromatography (GPC) and sulphuric acid-impregnated silica gel and then spiked with ^13^C_12_-1,2,3,4-CDD (control and TCDD-exposed groups) or ^13^C_12_-2,4,6,8-BDF (control and other exposed groups). For liver samples, hydroxylated compounds in the alkaline phase were transformed back into the non-ionic form by acidification with sulphuric acid (pH 2), back-extracted twice with tBME/hexane, cleaned up using acetonitrile partitioning, and then derivatized into methoxylated forms using trimethylsilyldiazomethane (TMSD). The derivatized solutions were further cleaned up with GPC, spiked with ^13^C_12_-CB-170 (control and TCDD-exposed groups) or ^13^C_12_-BDE-126 (control and other exposed groups), and then stored in amber glass vials for instrumental analysis. The whole procedure was conducted under UV-cut lighting to prevent photolytic degradation of brominated compounds.

Instrumental analysis was conducted using a gas chromatograph (GC) (7890A, Agilent, USA) coupled with a quadrupole mass spectrometer (MS) (5975C, Agilent Technologies, Santa Clara, CA, USA) in election ionization, selective ion monitoring mode. The GC column was a DB-5ht (0.25 mm × 0.1 µm × 15 m, Agilent Technologies, Santa Clara, CA, USA). The injector port and transfer line temperatures were 280 and 290 °C, respectively. The oven temperature program for the parent compounds was: 160 °C (hold 1 min), 10 °C/min to 200 °C, 5 °C/min to 240 °C, and then 40 °C/min to 310 °C (hold 3 min); and for methoxylated compounds: 110 °C (hold 1 min), 10 °C/min to 210 °C, 5 °C/min to 240 °C, and then 40 °C/min to 310 °C (hold 1 min). The MS source temperature was 230 °C for chlorinated compounds and 280 °C for brominated compounds. TCDD, TrBDF, TeBDF, PeBDF, TrBCDF, and their dehalogenated products were monitored using the most two intense ions of the molecular ion clusters [M]^+^ and quantified based on the response factors of the authentic standards relative to the respective isotope-labelled surrogates. Due to the lack of authentic standards, monomethoxylated derivatives (MeO-TCDD, -TrBDF, -TrBCDF, -TeBDF, and -PeBDF) were identified based on the presence of both the [M]^+^ clusters and the demethylated fragment ion clusters [M−CH_3_]^+^ characteristic of methylated phenols [39]. Each [M]^+^ cluster was monitored using the three most intense ions and each [M−CH_3_]^+^ cluster was monitored using the two most intense ions. Potential peaks were identified as methoxylated derivatives if the intensity ratios within these ion clusters did not deviate more than 20% from the theoretical Cl/Br isotopic ratios. The detected methoxylated compounds were semi-quantified against the isotope-labelled surrogates, assuming a relative response factor of 1. Recoveries were 72% to 111% for ^13^C_12_-1,2,3,4-CDD, 79% to 128% for ^13^C_12_-2,4,6,8-BDF, and 50% to 68% for ^13^C_12_-6-OH-BDE-47. Concentrations were expressed on a wet weight basis. Validation of the sample pretreatment procedure using spiked corn oil samples showed that the analysed concentrations of TCDD, TeBDF, and PeBDF deviated only 11%, 15%, and 17% of the respective spiked values.

### 2.4. Calculation of Elimination Kinetic Parameters

The elimination kinetics of the administered compounds were investigated using the R statistical environment, version 4.3.2 [40]. First-order kinetics were fitted as linear models between log_10_-transformed concentration and time. Decreasing trends deviating from first-order kinetics (*R*^2^ < 0.95) were fitted as two-phase linear models using the R package PK [41] version 1.3.6. Statistical significance was assumed at *p*-values < 0.05.

## 3. Results

### 3.1. Uptake and Distribution in Liver, Brain, and Plasma

After exposure, the largest portions of the administered dioxin-related compounds were detected in the liver (Table 1). The hepatic uptake was the highest for TCDD (up to 84% of the administered dose), followed by 2,3,7,8-substituted dibenzofurans in decreasing order with the number of Br substitutions (42% for TrBCDF, 33% for TeBDF, and 29% for PeBDF), but was very low for TrBDF (<0.01%). The partitioning in the brain and plasma accounted for less than 0.5% of the respective doses, and the tissue distribution ratios were also in a similar order to that observed in the liver: TCDD > TrBCDF ≈ TBDF > PeBDF > TrBDF. The brain distribution ratios of 2,3,7,8-substituted PBDFs and TrBCDF were at least an order of magnitude lower than that for TCDD (Table 1), and the difference may be explained by the blood-to-brain transfer efficiency, as indicated by the brain:plasma concentration ratio. This ratio was the highest for TCDD and TrBDF (3.0 and 3.2 at 1 d post exposure, respectively) and decreased with subsequent Br substitutions: TrBCDF (0.60) > TeBDF (0.33) > PeBDF (0.19) (Table S1). These results suggest that the increasing molecular size of higher brominated dibenzofurans may affect their uptake efficiencies and impede their transfer to the brain.

### 3.2. Presence of Monohydroxylated Metabolites in Liver

**Figure 1:**
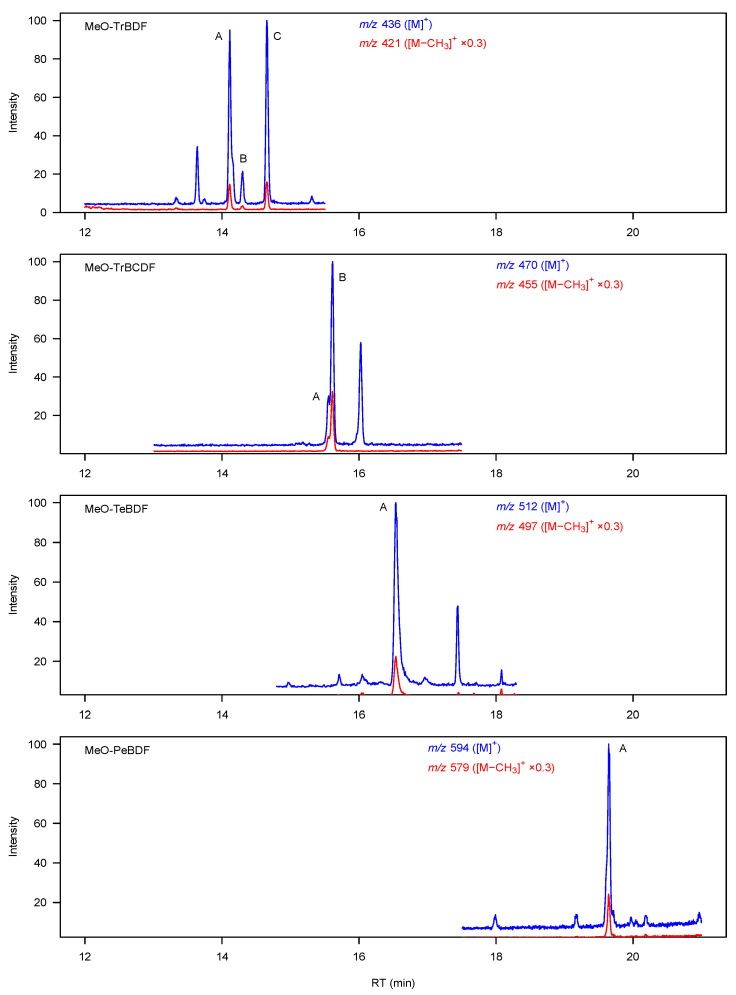
SIM chromatograms of monomethoxylated derivatives of TrBDF, TrBCDF, TeBDF, and PeBDF ([M]^+^ and [M−CH_3_]^+^, scaled with factors of 1 and 0.3, respectively) in the TMSD-treated phenolic phase extracts of pooled liver samples from exposed mice (*t* = 1 d). Peaks matching the theoretical isotopic ratios are marked with letters.

Monomethoxylated derivatives of TCDD (MeO-TCDDs) were not detected in the TMSD-treated phenolic fractions of the pooled liver extracts from TCDD-exposed mice, indicating the absence of monohydroxylated metabolites (OH-TCDDs) and negligible hepatic metabolism of TCDD via monooxygenation under the experimental conditions. In contrast, monohydroxylated metabolites were detected for all the tested dibenzofurans in the form of methoxylated derivatives present in the TMSD-treated phenolic fractions of liver extracts from mice exposed to the respective parent compounds (Figure 1). The numbers of peaks detected for MeO-TrBDF, MeO-TrBCDF, MeO-TeBDF, and MeO-PeBDF were 3, 2, 1, and 1, respectively. The extent of metabolic transformation to hydroxylated products can be evaluated through the molar ratio of metabolite vs. parent compound (M/P) in the liver (Table S1). TrBDF was the most extensively metabolised, as its M/P ratio at 1 d post exposure was 0.27, indicating that up to 21% of the TrBDF amount (M/(M + P) ratio) in the liver existed as hydroxylated metabolites. All 2,3,7,8-substituted dibenzofurans were much less metabolised, especially the highest brominated compound, PeBDF (M/P = 3.5 × 10^−5^ to 5.1 × 10^−5^). However, the higher M/P ratios observed for the tetrabrominated compound TeBDF (0.78 × 10^−3^ to 1.5 × 10^−3^) suggest a faster metabolism than for TrBCDF (0.83 × 10^−4^ to 4.5 × 10^−4^).

### 3.3. Elimination Kinetics in Liver, Brain, and Plasma

**Figure 2:**
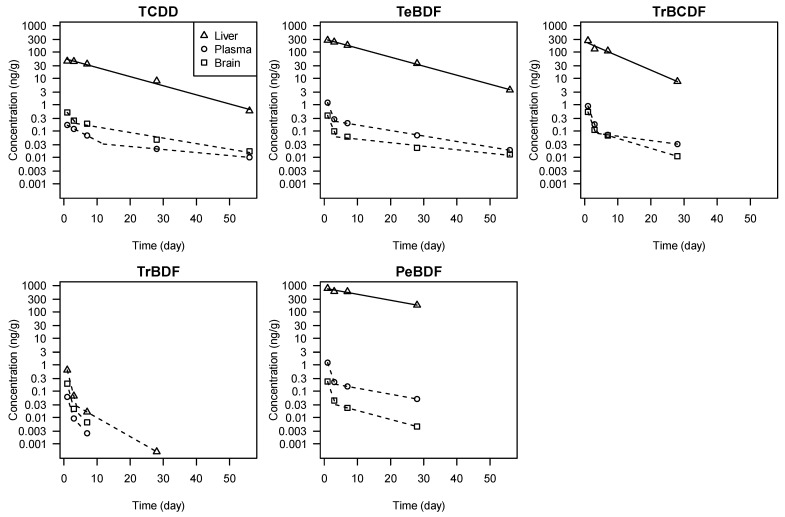
Time trends of TCDD, TrBDF, TeBDF, PeBDF, and TrBCDF concentrations in the liver, plasma, and brain of exposed mice. Solid lines represent fitted log–linear relationships (first-order kinetics), whereas dashed lines represent fitted two-phase kinetics as shown in Table 2.

After administration, the concentrations of TCDD, TrBCDF, TeBDF, and PeBDF in the liver decreased with time in log–linear relationships, thus obeying first-order kinetics (Figure 2, Table 2). The hepatic elimination half-time calculated for TCDD was 8.7 d. For dibenzofurans, the half-time increased with the number of Br substitutions: TrBDF (4.2 d in terminal phase) < TrBCDF (5.6 d) < TeBDF (8.8 d) < PeBDF (13 d) (Table 2).

The trends in the brain and plasma followed two-phase kinetics, as the decrease was generally faster between 1 and 3 d, then became slower after 7 d (Figure 2). However, the parameters of the elimination kinetics could not be evaluated reliably because of the limited number of data points available for two-phase model-fitting (Table 2). The calculated terminal half-times for the tetra-substituted compounds TCDD, TrBCDF, and TeBDF in these tissues were generally longer than in the liver, possibly due to their redistribution from major storage tissues such as the liver.

## 4. Discussion

The liver is known as the major storage tissue for 2,3,7,8-substituted PCDDs/PCDFs in various mammals including rodents [43,44], and the sequestration of dioxins by certain hepatic proteins has been well documented [45,46]. Our data indicate that 2,3,7,8-substituted brominated dibenzofurans also accumulate mainly in the liver, albeit with lower uptake ratios compared with those of TCDD. The extremely low retention of the non-2,3,7,8-substituted compound TrBDF in the liver of exposed mice can be explained by its rapid metabolism and/or lack of protein sequestration. As both molecular size and dose were found to have negative effects on uptake efficiency [47], the lower uptake of the brominated compounds relative to TCDD in the present study may have been caused not only by their increasing molecular size with the number of Br substitution but also by their higher doses. Previous studies on TCDD- and 2,3,7,8-tetrabromodibenzo-*p*-dioxin (TBDD)-exposed mice and rats also reported high accumulation in the liver and lower uptake ratios of TBDD compared with those of TCDD [47,48,49]. The lower dose of TCDD in our experiment may be a reason for its hepatic uptake ratio being higher than the average of 42.6% previously reported for C57BL/6N mice exposed to a high oral dose of 24 µg/kg bw [48]. At low subcutaneous doses, Nagao et al. [49] found the uptake of TBDD (0.6 µg/kg bw) and TCDD (0.3 µg/kg bw) in rat liver to be comparable (39% and 46% on day 3 after exposure, respectively), despite the difference in molecular size.

The low distribution ratios to the brain of exposed mice (Table 1) are consistent with previous results reported by Ishida et al. [50], who found that the brains of rats orally exposed to TCDD contained 0.01%–0.02% of the dose. Understanding the brain transfer efficiencies of dioxin-like compounds is important for interpreting their potential neurotoxicity. Kimura et al. [23,33] reported behavioural toxicity in mice exposed to TCDD, TeBDF, or TrBCDF during the developmental stage but not in those exposed to PeBDF or TrBDF, and the absence of the effect may reflect the very low amounts of PeBDF and TrBDF transferred to the brain observed in the present study.

Hydroxylated metabolites of TCDD have been detected in exposure experiments with rats [51] and more metabolically capable species such as dogs [52], and in a study on a TCDD-poisoned human patient [53]. The absence of OH-TCDD at detectable levels in the liver of TCDD-exposed mice in our experiment indicates that TCDD was less metabolisable than brominated dibenzofurans at similar TEQ levels. Previous studies on hydroxylated metabolites of 2,3,7,8-tetrachlorodibenzofuran (TeCDF) in rats [35,54] showed that the unsubstituted positions of the C–C bonds adjacent to the ether linkage are favourable hydroxylation sites. Thus, the most plausible hydroxylated metabolites of TrBDF, TrBCDF, and TeBDF may be 4-/6-/7-OH-TrBDFs, 4-/6-OH-TrBCDFs, and 4-OH-TeBDF, respectively (Figure S1), and their numbers are identical to the numbers of metabolites found in the liver of exposed mice (Figure 1). However, the only PeBDF metabolite detected may not have the OH group at positions 4 or 6, as an earlier study on metabolites of 1,2,3,7,8-pentachlorodibenzofuran (PeCDF) in rats also found a single OH-PeCDF, which was likely a NIH-shift product [55]. Finally, the extensive metabolism of the non-2,3,7,8-substituted compound TrBDF confirms that the presence of four halogens at the 2, 3, 7, and 8 positions of the dibenzofuran structure is a major factor for the biological persistence of halogenated dibenzofurans.

The elimination half-time of TCDD from the liver of exposed mice in the present study (8.7 d) was in good agreement with the data obtained by monitoring the total radioactivity of unmetabolised and metabolised ^3^H-TCDD in the liver of C57BL/6J mice (7.1–8.5 d [31]). For brominated dibenzofurans, the increase in hepatic half-times with the number of Br substitutions was consistent with the trend observed for tetra- and pentachlorinated dibenzofurans in rats [45]. The half-time of TeBDF was very similar to that of TCDD (Table 2) and much longer than the value reported for TeCDF in the liver of C57BL/6J mice (1.9 d [32]). A slower hepatic elimination of TeBDF compared with TeCDF was also reported for Wistar rats [27]. Similarly, PeBDF may be hepatically more persistent than PeCDF, as the latter has been reported to have a much shorter elimination half-time than that of TCDD in the liver of Sprague-Dawley rats [45]. Possible causes for the slower hepatic elimination of PBDFs compared with their chlorinated analogues, PCDFs, may be stronger effects of the larger Br substitutions in hindering metabolism and/or enhancing affinity with liver proteins. These effects may vary with the substitution position, as TeBDF had a higher metabolism ratio than TrBCDF (Section 3.2), but its elimination was slower. Considering the very low conversion ratios to monohydroxylated metabolites and the half-times of the parent compounds relative to TCDD, the main elimination route of 2,3,7,8-substituted dibenzofurans from mouse liver may have been passive portioning rather than metabolism.

Elimination kinetics with a rapid initial phase and a gradual terminal phase are commonly associated with multi-compartmental systems and correspond to distribution to various body compartments and subsequent steady-state elimination [56]. The presence of a faster distribution phase in the brain and plasma of exposed mice (Figure 2) indicates that the tested dioxin-like compounds are not specifically retained in these tissues, unlike in the liver where they can be sequestered by liver proteins. TrBDF followed two-phase kinetics in all the investigated mouse tissues, consistent with its poor retention even in the liver.

A two-phase kinetic was also observed for OH-TrBCDFs in the liver, suggesting a relatively rapid distribution of these TrBCDF metabolites to other tissues. OH-TeBDF and OH-PeBDF were not readily distributed, as their elimination kinetics in the liver were consistent with those of their parent compounds, with similar half-times (Table 2) and no significant change in M/P ratios with time. It is important to note that the elimination rates and half-times observed for all metabolites in the present study do not represent their intrinsic toxicokinetics because the tissue concentrations of the metabolites were dependent on those of their parent compounds.

The present study had several limitations. The purity of PeBDF and TrBCDF was only 77%, and the presence of impurities might influence metabolism. However, such influence may have been insignificant, as the metabolite/parent ratios were consistent across all brominated dibenzofurans. Our data set had only 4–5 time points for each compound, limiting the reliability of more complex two-phase kinetic models. Finally, the detected monohydroxylated metabolites were only semi-quantified, and their substitution positions could not be determined due to the nonavailability of authentic standards.

## 5. Conclusions

This study confirmed that the substitution by bromine/chlorine at all 2,3,7,8 positions of the dibenzofuran molecular structure is important for the retention and metabolic stability of PBDFs and PXDFs in animal tissues. 2,3,7,8-substituted PBDFs and PXDFs accumulated substantially in the liver of exposed mice, and their presence in the brain was reported for the first time. The hepatic uptake of TrBCDF, TeBDF, and PeBDF was two to three times lower compared to TCDD, and their brain transfer was also less efficient, probably due to hindrance by the increasing molecular size with a higher number of bromine substitutions. However, 2,3,7,8-substituted dibenzofurans and TCDD were comparably persistent in the mouse liver, despite the former being easier to metabolise to monohydroxylated products. Considering the slow elimination of TrBCDF, TeBDF, and PeBDF, higher toxic equivalency factors may need to be used for the risk assessment of 2,3,7,8-substituted PBDFs and PXDFs instead of the current interim values based on the less persistent PCDF analogues.

## Figures and Tables

**Table 1:** Distribution of TCDD, TrBDF, TeBDF, PeBDF, and TrBCDF in the liver, plasma, and brain of exposed mice (as fractions of the administered doses).

Compound	Day	Liver	Plasma ^1^	Brain
TCDD	1	0.69	3.3 × 10^−3^	3.6 × 10^−3^
	3	0.84	2.4 × 10^−3^	1.8 × 10^−3^
	7	0.73	1.5 × 10^−3^	1.4 × 10^−3^
	28	0.16	4.8 × 10^−4^	3.4 × 10^−4^
	56	0.012	2.7 × 10^−4^	1.2 × 10^−4^
TrBDF	1	8.0 × 10^−5^	9.6 × 10^−6^	1.1 × 10^−5^
	3	9.1 × 10^−6^	1.4 × 10^−6^	1.1 × 10^−6^
	7	2.3 × 10^−6^	4.1 × 10^−7^	3.7 × 10^−7^
	28	7.9 × 10^−7^	ND ^2^	ND ^2^
TrBCDF	1	0.42	1.7 × 10^−3^	3.4 × 10^−4^
	3	0.24	3.5 × 10^−4^	7.2 × 10^−5^
	7	0.23	1.4 × 10^−4^	4.5 × 10^−5^
	28	0.015	7.4 × 10^−5^	7.5 × 10^−6^
TeBDF	1	0.33	1.7 × 10^−3^	1.9 × 10^−4^
	3	0.31	3.8 × 10^−4^	5.0 × 10^−5^
	7	0.26	2.8 × 10^−4^	3.0 × 10^−5^
	28	0.053	1.1 × 10^−4^	1.1 × 10^−5^
	56	5.2 × 10^−3^	3.2 × 10^−5^	6.4 × 10^−6^
PeBDF	1	0.29	5.4 × 10^−4^	3.6 × 10^−5^
	3	0.25	9.9 × 10^−5^	6.5 × 10^−6^
	7	0.27	6.9 × 10^−5^	3.7 × 10^−6^
	28	0.085	2.6 × 10^−5^	7.1 × 10^−7^

^1^ calculated from plasma concentrations assuming a plasma/body weight ratio of 6% for mouse [42]; ^2^ not detected

**Table 2:** Elimination rates (log_10_-based) and half-times of TCDD, TrBDF, TeBDF, PeBDF, TrBCDF, and their hydroxylated metabolites in the tissues of exposed mice. The 95% confidence intervals for first-order kinetics are shown in parenthesis.

Tissue	Compound	Model	Elimination Rate (d^−1^)	Half-Time (d)	Concentration at *t* = 0 (ng/g)	*R* ^2^
Liver	TCDD	First-order	−0.0346 (±0.0058)	8.7 (7.4–10)	57 (39–84)	0.992
	TrBDF	Two-phase *	−0.493, −0.072	0.61, 4.2	2.0	
	TrBCDF	First-order	−0.0542 (±0.0214)	5.6 (4.0–9.2)	250 (120–510)	0.983
	TeBDF	First-order	−0.0343 (±0.0017)	8.8 (8.4–9.2)	310 (280–350)	0.999
	PeBDF	First-order	−0.0227 (±0.0089)	13 (9.5, 22)	790 (580–1100)	0.984
	OH-TrBDF	Two-phase *	−0.555, −0.0262	1.2, 11	0.65	
	OH-TrBCDF	Two-phase *	−0.418, −0.033	0.72, 9.2	0.34	
	OH-TeBDF	First-order	−0.0369 (±0.0085)	8.1 (6.6–11)	0.38 (0.22–0.65)	0.985
	OH-PeBDF	First-order	−0.0262 (±0.0179)	11 (6.8–36)	0.036 (0.020–0.065)	0.952
Plasma	TCDD	Two-phase	−0.0657, −0.0115	4.6, 26	0.19	0.999
	TrBDF	Two-phase *	−0.410, −0.140	0.73, 2.1	0.15	
	TrBCDF	Two-phase *	−0.347, −0.0168	0.87, 18	2.0	
	TeBDF	Two-phase	−0.316, −0.0208	0.95, 14	2.5	0.999
	PeBDF	Two-phase *	−0.368, −0.0227	0.82, 13	2.8	
Brain	TCDD	Two-phase	−0.155, −0.0211	1.9, 14	0.73	0.995
	TrBDF	Two-phase *	−0.478, −0.127	0.63, 2.4	0.57	
	TrBCDF	Two-phase *	−0.341, −0.0377	0.88, 8.0	1.2	
	TeBDF	Two-phase	−0.300, −0.0134	1.0, 22	0.78	0.999
	PeBDF	Two-phase *	−0.359, −0.0337	0.84, 8.9	0.53	

* elimination rate calculated directly from the only two data points available for each phase, *R*^2^ not calculated.

## Data Availability

The original contributions presented in the study are included in the article/Supplementary Material, further inquiries can be directed to the corresponding author.

## Supplementary Materials

The following supporting information can be downloaded at: https://www.mdpi.com/article/10.3390/toxics12090656/s1, Figure S1: Plausible hydroxylated metabolites of TrBDF, TrBCDF, and TeBDF; Table S1: Details on the mouse liver, plasma, and brain samples and concentrations of TCDD, TrBDF, TeBDF, PeBDF, and TrBCDF.

## References

[1] Persson L., Almroth B.M.C., Collins C.D., Cornell S., de Wit C.A., Diamond M.L., Fantke P., Hassellöv M., MacLeod M., Ryberg M.W. (2022). Outside the safe operating space of the planetary boundary for novel entities. Environ. Sci. Technol..

[2] Richardson K., Steffen W., Lucht W., Bendtsen J., Cornell S.E., Donges J.F., Drüke M., Fetzer I., Bala G., von Bloh W. (2023). Earth beyond six of nine planetary boundaries. Sci. Adv..

[3] DeVito M., Bokkers B., van Duursen M.B.M., van Ede K., Feeley M., Antunes Fernandes Gáspár E., Haws L., Kennedy S., Peterson R.E., Hoogenboom R. (2024). The 2022 World Health Organization reevaluation of human and mammalian toxic equivalency factors for polychlorinated dioxins, dibenzofurans and biphenyls. Regul. Toxicol. Pharmacol..

[4] Nishijo M., Pham T.T., Nguyen A.T.N., Tran N.N., Nakagawa H., Hoang L.V., Morikawa Y., Ho M.D., Kido T., Nguyen M.N. (2014). 2,3,7,8-Tetrachlorodibenzo-*p*-dioxin in breast milk increases autistic traits of 3-year-old children in Vietnam. Mol. Psychiatry.

[5] Viluksela M., Pohjanvirta R. (2019). Multigenerational and transgenerational effects of dioxins. Int. J. Mol. Sci..

[6] Weber R., Kuch B. (2003). Relevance of BFRs and thermal conditions on the formation pathways of brominated and brominated–chlorinated dibenzodioxins and dibenzofurans. Environ. Int..

[7] Altarawneh M., Saeed A., Al-Harahsheh M., Dlugogorski B.Z. (2019). Thermal decomposition of brominated flame retardants (BFRs): Products and mechanisms. Prog. Energy Combust. Sci..

[8] Hanari N., Kannan K., Okazawa T., Kodavanti P.R.S., Yamashita N. (2006). Occurrence of polybrominated biphenyls, polybrominated dibenzo-*p*-dioxins, and polybrominated dibenzofurans as impurities in commercial polybrominated diphenyl ether mixtures. Environ. Sci. Technol..

[9] Kajiwara N., Noma Y., Takigami H. (2008). Photolysis studies of technical decabromodiphenyl ether (DecaBDE) and ethane (DeBDethane) in plastics under natural sunlight. Environ. Sci. Technol..

[10] Suzuki G., Matsukami H., Michinaka C., Hashimoto S., Nakayama K., Sakai S. (2021). Emission of dioxin-like compounds and flame retardants from commercial facilities handling Deca-BDE and their downstream sewage treatment plants. Environ. Sci. Technol..

[11] Tasaki T., Takasuga T., Osako M., Sakai S. (2004). Substance flow analysis of brominated flame retardants and related compounds in waste TV sets in Japan. Waste Manag..

[12] Sindiku O., Babayemi J.O., Tysklind M., Osibanjo O., Weber R., Watson A., Schlummer M., Lundstedt S. (2015). Polybrominated dibenzo-*p*-dioxins and dibenzofurans (PBDD/Fs) in e-waste plastic in Nigeria. Environ. Sci. Pollut. Res..

[13] Ma J., Addink R., Yun S., Cheng J., Wang W., Kannan K. (2009). Polybrominated dibenzo-*p*-dioxins/dibenzofurans and polybrominated diphenyl ethers in soil, vegetation, workshop-floor dust, and electronic shredder residue from an electronic waste recycling facility and in soils from a chemical industrial complex in Eastern China. Environ. Sci. Technol..

[14] Takahashi S., Tue N.M., Takayanagi C., Tuyen L.H., Suzuki G., Matsukami H., Viet P.H., Kunisue T., Tanabe S. (2017). PCBs, PBDEs and dioxin-related compounds in floor dust from an informal end-of-life vehicle recycling site in northern Vietnam: Contamination levels and implications for human exposure. J. Mater. Cycles Waste Manag..

[15] Suzuki G., Someya M., Takahashi S., Tanabe S., Sakai S., Takigami H. (2021). Dioxin-like activity in Japanese indoor dusts evaluated by means of in vitro bioassay and instrumental analysis: Brominated dibenzofurans are an important contributor. Environ. Sci. Technol..

[16] Tue N.M., Suzuki G., Takahashi S., Kannan K., Takigami H., Tanabe S. (2013). Dioxin-related compounds in house dust from New York State: Occurrence, in vitro toxic evaluation and implications for indoor exposure. Environ. Pollut..

[17] Tue N.M., Matsushita T., Goto A., Itai T., Asante K.A., Obiri S., Mohammed S., Tanabe S., Kunisue T. (2019). Complex mixtures of brominated/chlorinated diphenyl ethers and dibenzofurans in soils from the Agbogbloshie e-waste site (Ghana): Occurrence, formation, and exposure implications. Environ. Sci. Technol..

[18] Hashimoto S., Matsukami H., Ieda T., Suzuki G. (2021). Comprehensive screening of polybromochlorodibenzo-*p*-dioxins, dibenzofurans as mixed halogenated compounds in wastewater samples from industrial facilities by GC×GC/ToFMS and post-data processing. Chemosphere.

[19] Behnisch P.A., Hosoe K., Sakai S. (2003). Brominated dioxin-like compounds: In vitro assessment in comparison to classical dioxin-like compounds and other polyaromatic compounds. Environ. Int..

[20] Olsman H., Engwall M., Kammann U., Klempt M., Otte J., van Bavel B., Hollert H. (2007). Relative differences in aryl hydrocarbon receptor-mediated response for 18 polybrominated and mixed halogenated dibenzo-*p*-dioxins and -furans in cell lines from four different species. Environ. Toxicol. Chem..

[21] Samara F., Gullett B.K., Harrison R.O., Chu A., Clark G.C. (2009). Determination of relative assay response factors for toxic chlorinated and brominated dioxins/furans using an enzyme immunoassay (EIA) and a chemically-activated luciferase gene expression cell bioassay (CALUX). Environ. Int..

[22] Birnbaum L.S., Morrissey R., Harris M.W. (1991). Teratogenic effects of 2,3,7,8-tetrabromodibenzo-*p*-dioxin and three polybrominated dibenzofurans in C57BL/6N mice. Toxicol. Appl. Pharmcol..

[23] Kimura E., Suzuki G., Uramaru N., Endo T., Maekawa F. (2020). Behavioral impairments in infant and adult mouse offspring exposed to 2,3,7,8-tetrabromodibenzofuran in utero and via lactation. Environ. Int..

[24] Nakayama K., Tue N.M., Fujioka N., Tokusumi H., Goto A., Uramaru N., Suzuki G. (2022). Determination of the relative potencies of brominated dioxins for risk assessment in aquatic environments using the early-life stage of Japanese medaka. Ecotoxicol. Environ. Saf..

[25] van den Berg M., Denison M.S., Birnbaum L.S., DeVito M.J., Fiedler H., Falandysz J., Rose M., Schrenk D., Safe S., Tohyama C. (2013). Polybrominated dibenzo-*p*-dioxins, dibenzofurans, and biphenyls: Inclusion in the toxicity equivalency factor concept for dioxin-like compounds. Toxicol. Sci..

[26] van den Berg M., Birnbaum L.S., Denison M.S., DeVito M.J., Farland W., Feeley M., Fiedler H., Hakansson H., Hanberg A., Haws L. (2006). The 2005 World Health Organization reevaluation of human and mammalian toxic equivalency factors for dioxins and dioxin-like compounds. Toxicol. Sci..

[27] Golor G., Yamashita K., McLachlan M., Hutzinger O., Neubert D. (1993). Comparison of the kinetics of chlorinated and brominated dioxins and furans in the rat. Organohalog. Compd..

[28] Schulz T., Golor G., Körner W., Hagenmaier H., Neubert D. (1993). Comparative study on enzyme induction and tissue distribution of 2,3,7,8-tetrachlorodibenzo-*p*-dioxin, 2,3,4,7,8-pentachlorodibenzofuran and 2,3,4,7,8-pentabromodibenzofuran in marmoset monkeys (*Callithrix jacchus*). Organohalog. Compd..

[29] Kedderis L.B., Jackson J.A., Patterson D.G., Grainger J., Diliberto J.J., Birnbaum L.S. (1994). Chemical characterization and disposition studies with 1,2,7,8-tetrabromodibenzofuran in the rat. J. Toxicol. Environ. Health.

[30] Arnoldsson K., Haldén A.N., Norrgren L., Haglund P. (2012). Retention and maternal transfer of environmentally relevant polybrominated dibenzo-*p*-dioxins and dibenzofurans, polychlorinated dibenzo-*p*-dioxins and dibenzofurans, and polychlorinated biphenyls in zebrafish (*Danio rerio*) after dietary exposure. Environ. Toxicol. Chem..

[31] Birnbaum L.S. (1986). Distribution and excretion of 2,3,7,8-tetrachlorodibenzo-*p*-dioxin in congenic strains of mice which differ at the Ah locus. Drug Metab. Dispos..

[32] Decad G.M., Birnbaum L.S., Matthews H.B. (1981). Distribution and excretion of 2,3,7,8-tetrachlorodibenzofuran in C57BL/6J and DBA/2J mice. Toxicol. Appl. Pharmcol..

[33] Kimura E., Suzuki G., Uramaru N., Kakeyama M., Maekawa F. (2023). 2-Chloro-3,7,8-tribromodibenzofuran as a new environmental pollutant inducing atypical ultrasonic vocalization in infant mice. Toxicol. Res..

[34] Kimura E., Suzuki G., Uramaru N., Kakeyama M., Maekawa F. (2022). Liver-specific decrease in *Tff3* gene expression in infant mice perinatally exposed to 2,3,7,8-tetrabromodibenzofuran or 2,3,7,8-tetrachlorodibenzo-*p*-dioxin. Appl. Toxicol..

[35] Olson J.R., McGarrigle B.P., Gigliotti P.J., Kumar S., McReynolds J.H. (1994). Hepatic uptake and metabolism of 2,3,7,8-tetrachlorodibenzo-*p*-dioxin and 2,3,7,8-tetrachlorodibenzofuran. Fundam. Appl. Toxicol..

[36] Sovocool G.W., Mitchum R.K., Donnelly J.R. (1987). Use of the ‘ortho effect’ for chlorinated biphenyl and brominated biphenyl isomer identification. Biomed. Environ. Mass. Spectrom..

[37] Eguchi A., Nomiyama K., Devanathan G., Subramanian A., Bulbule K.A., Parthasarathy P., Takahashi S., Tanabe S. (2012). Different profiles of anthropogenic and naturally produced organohalogen compounds in serum from residents living near a coastal area and e-waste recycling workers in India. Environ. Int..

[38] Eguchi A., Nomiyama K., Ochiai M., Mizukawa H., Nagano Y., Nakagawa K., Tanaka K., Miyagawa H., Tanabe S. (2014). Simultaneous detection of multiple hydroxylated polychlorinated biphenyls from a complex tissue matrix using gas chromatography/isotope dilution mass spectrometry. Talanta.

[39] Nomiyama K., Tanizaki T., Ishibashi H., Arizono K., Shinohara R. (2005). Production mechanism of hydroxylated PCBs by oxidative degradation of selected PCBs using TiO_2_ in water and estrogenic activity of their intermediates. Environ. Sci. Technol..

[40] R Core Team (2023). R: A Language and Environment for Statistical Computing.

[41] Jaki T., Wolfsegger M.J. (2011). Estimation of pharmacokinetic parameters with the R package PK. Pharm. Stat..

[42] Riches A.C., Sharp J.G., Thomas D.B., Smith S.V. (1973). Blood volume determination in the mouse. J. Physiol..

[43] Weber L.W.D., Ernst S.W., Stahl B.U., Rozman K. (1993). Tissue distribution and toxicokinetics of 2,3,7,8-tetrachlorodibenzo-*p*-dioxin in rats after intravenous injection. Fundam. Appl. Toxicol..

[44] van den Berg M., DeJongh J., Poiger H., Olson J.R. (1994). The toxicokinetics and metabolism of polychlorinated dibenzo-*p*-dioxins (PCDDs) and dibenzofurans (PCDFs) and their relevance for toxicity. Crit. Rev. Toxicol..

[45] DeVito M.J., Ross D.G., Dupuy A.E., Ferrario J., McDaniel D., Birnbaum L. (1998). Dose–response relationships for disposition and hepatic sequestration of polyhalogenated dibenzo-*p*-dioxins, dibenzofurans, and biphenyls following subchronic treatment in mice. Toxicol. Sci..

[46] van Ede K.I., Aylward L.L., Andersson P.L., van den Berg M., van Duursen M.B.M. (2013). Tissue distribution of dioxin-like compounds: Potential impacts on systemic relative potency estimates. Toxicol. Lett..

[47] Kedderis L.B., Diliberto J.J., Jackson J.A., Linko P., Goldstein J.A., Birnbaum L.S. (1992). Effects of dose and route of exposure on dioxin disposition. Chemosphere.

[48] Abbott B.D., Birnbaum L.S., Diliberto J.J. (1996). Rapid distribution of 2,3,7,8-tetrachlorodibenzo-*p*-dioxin (TCDD) to embryonic tissues in C57BL/6N mice and correlation with palatal uptake in vitro. Toxicol. Appl. Pharmcol..

[49] Nagao T., Yamashita K., Golor G., Bittmann H., Körner W., Hagenmaier H., Neubert D. (1996). Tissue distribution after a single subcutaneous administration of 2,3,7,8-tetrabromodibenzo-*p*-dioxin in comparison with toxicokinetics of 2,3,7,8-tetrachlorodibenzo-*p*-dioxin in female Wistar rats. Life Sci..

[50] Ishida T., Matsumoto Y., Takeda T., Koga T., Ishii Y., Yamada H. (2010). Distribution of ^14^C-2,3,7,8-tetrachlorodibenzo-*p*-dioxin to the brain and peripheral tissues of fetal rats and its comparison with adults. J. Toxicol. Sci..

[51] Poiger H., Schlatter C. (1979). Biological degradation of TCDD in rats. Nature.

[52] Poiger H., Buser H.R., Weber H., Zweifel U., Schlatter C. (1982). Structure elucidation of mammalian TCDD-metabolites. Experentia.

[53] Sorg O., Zennegg M., Schmid P., Fedosyuk R., Valikhnovskyi R., Gaide O., Kniazevych V., Saurat J.H. (2009). 2,3,7,8-tetrachlorodibenzo-*p*-dioxin (TCDD) poisoning in Victor Yushchenko: Identification and measurement of TCDD metabolites. Lancet.

[54] Burka L.T., McGown S.R., Tomer K.B. (1990). Identification of the biliary metabolites of 2,3,7,8-tetrachlorodibenzofuran in the rat. Chemosphere.

[55] Pluess N., Poiger H., Schlatter C., Buser H.R. (1987). The metabolism of some pentachlorodibenzofurans in the rat. Xenobiotica.

[56] Lee M.L., Poon W.Y., Kingdon H.S. (1990). A two-phase linear regression model for biologic half-life data. J. Lab. Clin. Med..

