# Supplementary material for: Uptake, Elimination and Metabolism of Brominated Dibenzofurans in Mice
Source: Toxics. 2024 Sep 7;12(9):656. doi: 10.3390/toxics12090656 (PMC11435657; doi:10.3390/toxics12090656)
Supplement: Supplementary file 1 [file toxics-12-00656-s001.zip › toxics-3159533-supplementary.pdf]

Uptake, elimination and metabolism of brominated dibenzofurans in mice

Supplementary materials

Nguyen Minh Tue [1], Eiki Kimura [2,3], Fumihiko Maekawa [2], Akitoshi Goto [1], Naoto Uramaru [4,5], Tatsuya Kunisue [1], Go Suzuki [6]

[1] Center for Marine Environmental Studies (CMES), Ehime University, 2-5 Bunkyo-cho, Matsuyama 790-8577, Japan

[2] Health and Environmental Risk Division, National Institute for Environmental Studies (NIES), 16-2 Onogawa, Tsukuba 305-8506, Japan

[3] Department of Environmental Health, School of Medical Sciences, University of Fukui, 23-3 Matsuoka Shimoaizuki, Eiheiji 910-1193, Japan

[4] Division of Pharmaceutical Health Biosciences, Nihon Pharmaceutical University, 10281 Komuro, Ina-machi, Kitaadachi, Saitama 362-0806, Japan

[5] School of Health and Social Services, Center for University-wide Education, Saitama Prefectural University, 820 San-Nomiya, Koshigaya, Saitama 343-8540, Japan

[6] Material Cycles Division, NIES, 16-2 Onogawa, Tsukuba 305-8506, Japan

[*] Corresponding author. Phone/Fax: +81 89 927 8173; e-mail: tuenm@vnu.edu.vn

Table S1. Details on the mouse liver, plasma, and brain samples and concentrations of TCDD, TrBDF, TeBDF, PeBDF, and TrBCDF. Values in square braces are total concentrations of the respective monomethoxylated metabolites and molar ratios relative to the parent compounds.

t (day)	*n*	Dose	Body weight (g)		Tissue weight (g)			Concentration in pooled sample (ng/g)		
		(ng/ng)	at 0 day	at *t* day	Liver	Plasma (pooled)	Brain	Liver	Plasma	Brain
Control										
0	6	vehicle	18.2		0.601	1.094	0.442	ND[a]	ND[b]	ND[c]
			22.8		1.074		0.475			
			21.5		1.170		0.459			
			21.0		0.760		0.441			
			21.7		1.094		0.462			
			23.2		0.976		0.462			
TCDD										
1	5	3.0	21.3	21.5	1.012	1.299	0.462	45	0.17	0.51
			20.6	20.4	0.807		0.460	[ND]		
			22.0	21.9	1.082		0.457			
			20.9	21.2	1.039		0.461			
			23.1	23.1	1.008		0.456			
3	5	3.0	20.4	20.8	1.113	1.135	0.466	44	0.12	0.25
			21.7	22.1	1.193		0.452	[ND]		
			21.5	22.3	1.191		0.469			
			20.1	20.7	1.163		0.445			
			21.5	21.5	1.346		0.463			
7	6	3.0	23.2	24.2	1.493	1.409	0.467	35	0.068	0.19
			19.1	19.8	1.160		0.445	[ND]		
			21.2	22.1	1.324		0.452			
			21.1	23.4	1.192		0.488			
			20.3	21.6	1.658		0.435			
			20.5	21.7	1.152		0.461			
28	6	3.0	21.5	25.3	1.423	1.031	0.463	8.0	0.021	0.047
			22.2	25.8	1.253		0.487	[ND]		
			20.6	24.1	1.281		0.459			
			21.7	24.6	1.117		0.474			
			20.3	24.1	1.290		0.462			
			20.5	24.8	1.378		0.470			
56	6	3.0	19.5	24.0	1.074	1.317	0.454	0.58	0.010	0.017
			22.1	29.3	1.341		0.487	[ND]		
			20.6	26.0	1.313		0.466			
			21.5	28.3	1.312		0.477			
			21.5	29.1	1.399		0.470			
			20.9	25.3	1.355		0.463			
TrBDF										
1	5	378	22.0	21.9	1.066	1.125	0.476	0.63	0.060	0.19
			21.9	22.2	0.987		0.475	[0.18 (0.27)]		
			22.3	22.4	1.098		0.453			
			21.3	21.4	1.049		0.472			
			21.1	21.1	1.021		0.476			
3	6	378	24.6	24.3	1.310	1.628	0.469	0.063	0.0091	0.021
			22.5	22.4	1.212		0.453	[0.014 (0.21)]		
			24.0	23.5	1.206		0.475			
			22.4	22.4	1.286		0.470			
			24.2	24.0	1.256		0.474			
			23.8	23.9	1.256		0.474			
7	5	378	20.4	21.7	1.115	1.528	0.462	0.016	0.0025	0.0065
			22.3	24.3	1.418		0.453	[0.011 (0.64)]		
			21.3	22.6	1.214		0.463			
			22.2	22.6	1.164		0.472			
			21.6	22.6	1.223		0.446			
28	5	378	22.2	25.9	1.422	1.522	0.484	0.0005	<0.001	<0.001
			21.5	25.4	1.314		0.467	[<0.001]		
			22.4	26.3	1.335		0.484			
			22.4	26.5	1.285		0.474			
			22.6	26.5	1.309		0.498			
TeBDF										
1	5	45	20.8	20.8	1.167	1.352	0.457	280	1.2	0.39
			19.6	20.1	1.120		0.461	[0.45 (1.5×10^{-3})]		
			23.3	23.7	1.238		0.482			
			21.9	22.4	1.112		0.468			
			21.6	22.0	1.117		0.466			
3	5	45	20.9	21.2	1.250	1.316	0.461	240	0.28	0.098
			19.4	20.5	1.276		0.451	[0.22 (0.86×10^{-3})]		
			21.1	21.7	1.036		0.463			
			20.4	20.3	1.085		0.471			
			18.7	19.3	1.208		0.452			
7	6	45	20.5	21.7	1.197	1.285	0.463	180	0.20	0.061
			20.3	21.2	1.341		0.457	[0.18 (0.94×10^{-3})]		
			20.7	22.5	1.467		0.453			

			21.2	22.1	1.355		0.471			
			22.4	23.2	1.465		0.472			
			19.6	20.2	1.210		0.453			
28	6	45	21.3	26.1	1.484	1.739	0.447	37	0.069	0.023
			19.2	23.7	1.234		0.465	[0.045 (1.1×10^{-3})]		
			20.4	24.4	1.279		0.466			
			21.1	23.9	1.235		0.465			
			20.0	25.0	1.411		0.452			
			21.7	24.8	1.327		0.474			
56	6	45	22.4	27.1	1.405	1.960	0.482	3.6	0.019	0.013
			21.0	26.1	1.375		0.463	[0.0030 (0.78×10^{-3})]		
			23.1	29.5	1.483		0.480			
			19.7	25.3	1.271		0.467			
			22.8	29.1	1.432		0.474			
			21.9	28.0	1.471		0.477			
PeBDF										
1	5	135	20.3	20.5	1.009	1.310	0.456	780	1.2	0.23
			22.8	22.7	1.232		0.473	[0.042 (5.1×10^{-5})]		
			22.0	22.6	1.148		0.467			
			21.9	22.2	1.103		0.468			
			21.5	21.6	0.997		0.469			
3	6	135	23.2	23.6	1.406	1.742	0.466	600	0.22	0.044
			22.4	22.0	1.199		0.464	[0.025 (4.0×10^{-5})]		
			24.4	24.5	1.377		0.468			
			24.7	24.6	1.459		0.462			
			23.8	23.9	1.312		0.464			
			23.2	23.5	1.342		0.463			
7	6	135	22.3	23.7	1.373	1.644	0.476	590	0.15	0.023
			22.5	23.1	1.333		0.464	[0.022 (3.5×10^{-5})]		
			21.3	22.3	1.204		0.453			
			21.6	23.0	1.434		0.481			
			20.7	22.3	1.421		0.466			
			21.1	22.6	1.260		0.466			
28	5	135	22.7	26.9	1.596	1.699	0.485	180	0.050	<0.009
			23.6	27.3	1.217		0.488	[0.0068 (3.6×10^{-5})]		
			22.6	26.1	1.221		0.475			
			20.4	24.2	1.064		0.445			
			22.0	25.0	1.161		0.474			
TrBCDF										
1	5	31	23.7	22.7	1.144	1.307	0.470	270	0.89	0.53
			24.0	23.4	1.322		0.473	[0.13 (4.5×10^{-4})]		
			25.1	24.1	1.258		0.474			
			22.6	21.6	1.010		0.453			
			22.0	21.3	0.974		0.470			
3	5	31	22.3	21.9	1.273	1.204	0.467	130	0.18	0.11
			23.3	22.2	1.320		0.462	[0.019 (1.4×10^{-4})]		
			24.4	24.1	1.522		0.468			
			22.7	21.5	1.253		0.466			
			23.7	23.1	1.350		0.466			
7	5	31	23.5	25.4	1.687	1.486	0.472	110	0.072	0.068
			24.2	24.9	1.626		0.470	[0.0098 (0.83×10^{-4})]		
			23.4	23.9	1.505		0.461			
			22.1	22.6	1.360		0.467			
			21.9	22.0	1.342		0.463			
28	6	31	22.7	26.9	1.511	1.945	0.477	7.6	0.032	0.11
			20.7	24.8	1.322		0.465	[0.0020 (2.4×10^{-4})]		
			23.5	26.6	1.470		0.480			
			23.8	28.9	1.495		0.487			
			23.6	25.1	1.271		0.461			
			20.3	26.3	1.201		0.496			

[a] TCDD <0.003, TrBDF <0.0003, TeBDF <0.005, PeBDF <0.007, TrBCDF <0.002 ng/g

[b] TCDD <0.007, TrBDF <0.002, TeBDF <0.008, PeBDF <0.012, TrBCDF <0.004 ng/g

[c] TCDD <0.005, TrBDF <0.001, TeBDF <0.007, PeBDF <0.013, TrBCDF <0.002 ng/g

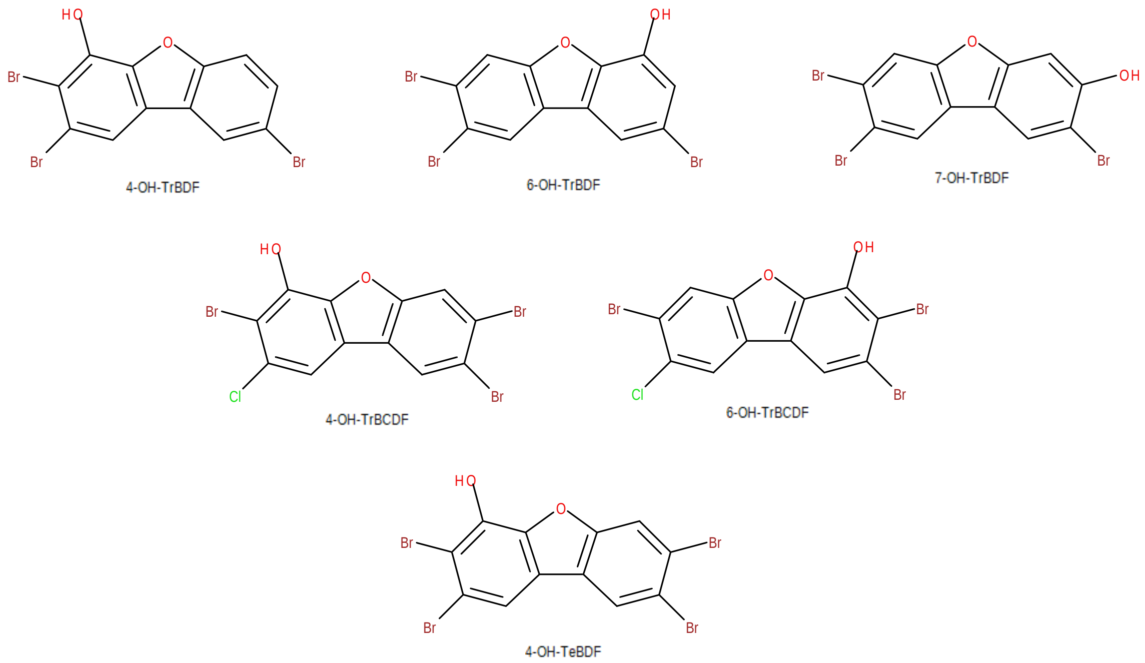

Figure S1. Plausible hydroxylated metabolites of TrBDF, TrBCDF, and TeBDF
